# The Positive Temperature Coefficient of Resistivity in BiFeO_3_ Films

**DOI:** 10.3390/nano12060892

**Published:** 2022-03-08

**Authors:** Qianqian Yang, Xiaolei Wang, Kaihua Yang, Jinxiang Deng, Ruijuan Nie, Qingsong Deng, Xuegang Chen, Hongwei Yang, Kailin Xu, Furen Wang

**Affiliations:** 1 Department of Physics and Optoelectronics Engineering, Faculty of Science, Beijing University of Technology, Beijing 100124, China; xiaoleiwang@bjut.edu.cn (X.W.); khy@bjut.edu.cn (K.Y.); jdeng@bjut.edu.cn (J.D.); xukl@emails.bjut.edu.cn (K.X.); 2 School of Physics, Peking University, Beijing 100871, China; rjnie@pku.edu.cn (R.N.); frwang@pku.edu.cn (F.W.); 3 Institute of Microstructure and Properties of Advanced Materials, Beijing University of Technology, Beijing 100124, China; qsdeng@bjut.edu.cn; 4 Institutes of Physical Science and Information Technology, Anhui University, Hefei 230601, China; xgchen@ahu.edu.cn

**Keywords:** BiFeO_3_, PTCR, superconductor, transport properties

## Abstract

The use of lead-free ceramic film materials with positive temperature coefficient of resistivity (PTCR) is widespread in temperature heaters and sensors in micro-electromechanical systems. In this research, the out of plane transport properties of the BiFeO_3_ (BFO) films have been studied. Surprisingly, PTCR was found in the BFO ceramic films due to the strongly correlated interaction between the multiferroic material BFO and the superconductor YBCO perovskite oxides. To our knowledge, this is the first report on the PTCR effect of BFO films. The BFO/YBCO interface and the bulk conductivity of BFO are important for the PTCR effect, as they make it possible to compare the transport properties of Au/BFO/YBCO- and YBCO/BFO/YBCO-type structures. PTCR was observed in Au/BFO/YBCO at a bias voltage of more than 2 V, but not in the YBCO/BFO/YBCO, even with a 40 V bias voltage. PTCR was found after BFO breakdown of a YBCO/BFO/YBCO capacitor. This indicated that the conductivity of BFO is critical for PTCR. The dependence of PTCR on the superconducting transition temperature illustrates that a cooper-pair can be injected into BFO. Our work presents a method by which to produce a lead-free ceramic film material with PTCR.

## 1. Introduction

BiFeO_3_ (BFO) with a perovskite structure is a multiferroic material with the coexistence of magnetic order and ferroelectric order at room temperature [1,2]. BFO is usually combined with ferromagnetic [3,4] and superconductor [5,6,7] materials, because of the novel physical properties induced by the interaction between spin, charge and orbital. In this study, the multiferroic/superconductor BFO/YBCO heterostructure were investigated. The anomalous transport property, i.e., PTCR (Positive temperature coefficient of resistivity), was found. PTCR refers the increase of the resistance with an increase of temperature. PTCR is very useful in commercial equipment, because it can be used in self-contained, power-limiting devices to prevent failure induced by thermal runaway, usually owing to the grain boundaries [8]. Additionally, multiferroic materials may be used to reduce the electromagnetic interference generated by the excessive use of high-frequency electronic devices [9]. There are now mainly two kinds of materials with PTCR characteristics, namely, barium titanate series bulk materials [10,11,12] and vanadium oxide series bulk materials [13,14], both of which represent mature technologies.

In addition to bulk materials, film materials with PTCR characteristics are also important. For example, these materials can be used in surface heaters in MEMS to command the heaters and temperature sensors. To date, PTCR has been observed in BaTiO_3_ [15] and Pb(Ti, Zr)O_3_ [16] films. PTCR was found in BaTiO_3_ films in both ferroelectric and paraelectric states. Heywang et al. reported that the Curie temperature of BaTiO_3_ in the Pt/BaTiO_3_/IrO_2_ heterojunction was about 250 K. However, the observed temperature of PTCR effect is higher than 300 K, so BaTiO_3_ is in paraelectric state [17]. The PTCR effect occurs in BaTiO_3_ films when the applied voltage is greater than 2 V, and becomes notable with increases in applied voltage [17]. Those authors attributed the PTCR to the decreasing dielectric constant of BaTiO_3_ thin films with an increase of temperature [17].

In addition, Yang et al. reported that in Pt/BaTiO_3_/YBCO heterojunctions, BaTiO_3_ films exhibit a PTCR effect in the ferroelectric state, and a PTCR effect exists in both forward and reverse voltages of Pt [18]. This effect was attributed to the increase of dielectric constant. However, in contrast to paraelectric BaTiO_3_ film, the authors proposed that the increase in dielectric constant with decreasing the temperature was the direct cause of the PTCR effect. Meanwhile, Heywang et al. think that the increase of dielectric constant leads to an increase in the interfacial barrier and then PTCR effect. The temperature range of the PTCR effect is not fixed. As for the Pb(Ti, Zr)O_3_ films, a PTCR effect exists in the Au/Pb(Ti, Zr)O_3_/Nb-SrTiO_3_ heterostructure. The temperature range of this effect is 50 K–240 K [19]. PTCR is considered to be caused by tunneling through the Pb(Ti, Zr)O_3_/Nb-SrTiO_3_ interface.

For multiferroic BFO, the PTCR effect was observed in BFO nanoparticles [20] and bulks [21,22,23]. However, to the best of our knowledge, PTCR in BFO thin films has not been reported. In this paper, we report the PTCR in the BFO films at different temperature ranges for different top electrodes, within which BFO thin films are in a ferroelectric state. We will clarify the transport mechanism and identify the origins of this PTCR effect.

## 2. Materials and Methods

**Figure 1:**
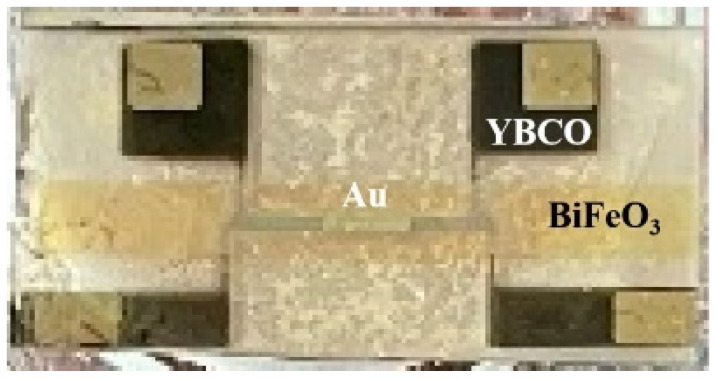
Image of the real Au/BFO/YBCO heterostructure.

Multilayer films were fabricated by pulsed laser deposition. The SrTiO_3_(001) single crystalline substrate, the BiFeO_3_ and the YBCO target employed in this work were produced by Hefei Kejing Materials Technology Co. Ltd. of China. A commercial KrF excimer laser (Lambda Physik LPX-300, Gottingen, Germany; 248 nm, 20 ns) was used as a light source. The films were prepared using a pulsed laser deposition system (Model LMBE 450; SKY Company, Shenyang, China). The pulsed laser deposition method has the advantages of consistency of film composition with target composition, easy control of deposition conditions, high deposition rate and so on. The fabrication conditions are described elsewhere [24]. An image of the real heterostructure is shown in Figure 1.

The crystal structure was investigated by X-ray diffraction (XRD) using Cu Ka radiation (λ = 1.54056 Å). TEM characterization was performed on a FEI Tecnai F30 (FEI, Hillsboro, OR, USA) scanning transmission electron microscope. Cross-section TEM samples were prepared by focused ion beam. The superconducting and transport properties of the YBCO films were characterized by the standard four-point method. Oxygen vacancies were studied using a MICROLAB MK II X-ray photoelectron spectroscope (XPS, VG company, East Grinstead, England). The sample was introduced into the XPS system within 1 h after being removed from the deposition system. The vacuum of the analysis chamber was less than 3 × 10^7^ Pa. Using a 1253.6 eV Mg Ka line, the X-ray source operated at 14.5 kV. All the binding energies were corrected for the sample charging effect with reference to the C 1s line at 284.6 eV.

## 3. Results and Discussion

### 3.1. Structures in the BFO Capacitors

**Figure 2:**
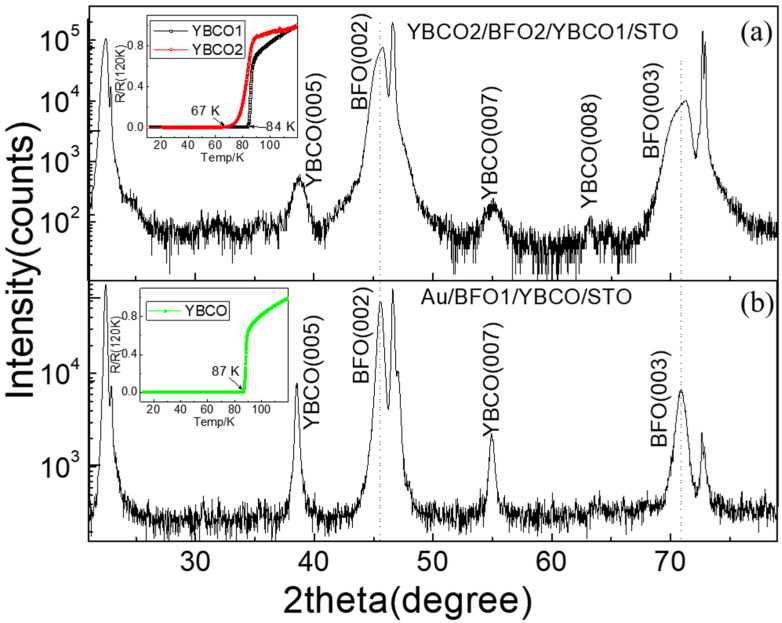
XRD images of the YBCO2/BFO2/YBCO1 capacitor (**a**) and the Au/BFO1/YBCO capacitor (**b**). The inset of (**a**) shows the resistive versus temperature curves of YBCO1 (black curve) and YBCO2 (red curve). The inset of (**b**) shows the resistive versus temperature curve of YBCO in the Au/BFO1/YBCO capacitor.

X-ray diffraction images of the YBCO2/BFO2/YBCO1 and Au/BFO1/YBCO capacitors are shown in Figure 2. The (001)-oriented diffraction peaks of YBCO and BFO may be clearly seen. No peaks of other phases are seen, indicating that the film underwent good crystallization. The BFO peaks in BFO2 shifted to the higher angle, exhibiting an increase of oxygen content compared to BFO1. The inserts of Figure 2 show the resistance versus temperature curves of YBCO1 and YBCO2 films. The superconducting transition temperature (T_C_) was 67 K for YBCO2, 84 K for YBCO1 in BFO2 capacitor and 87 K for YBCO in BFO1 capacitor.

**Figure 3:**
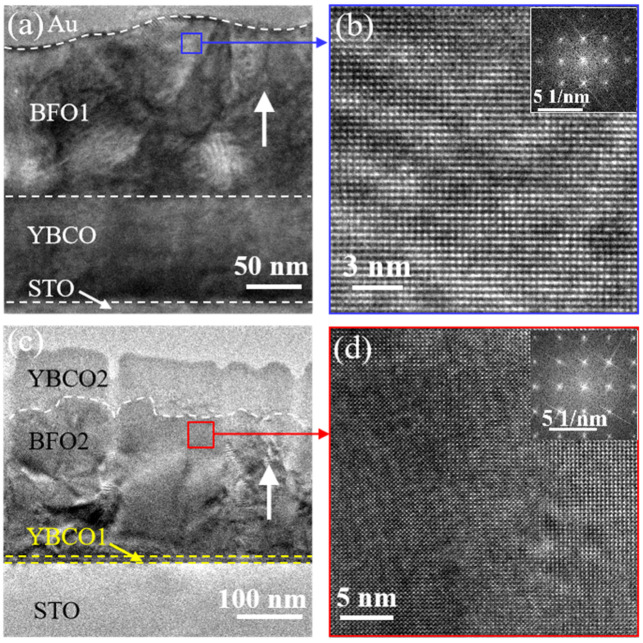
Cross section TEM images of the YBCO2/BFO2/YBCO1 capacitor (**a**) and the Au/BFO1/YBCO capacitor (**c**). The white upward arrows in (**a**,**c**) indicate the direction of growth. High resolution TEM images of the BFO films taken in the [110] crystallographic direction of BFO1 (**b**) and BFO2 (**d**). Figure 2b taken from [25].

TEM images of the two kinds of samples are shown in Figure 3, indicating the clear interface in the two BFO capacitors. According to the cross section TEM images, the thicknesses of films should be Au(40 nm)/BFO(150 nm)/YBCO(80 nm) and YBCO(70 nm)/BFO(220 nm)/YBCO(10 nm), respectively. Figure 3b, d show the HRTEM of the corresponding BFO in Figure 3a,c. The (001)-oriented growth of BFO may be observed. The FFT is shown in the insert of Figure 3b,d. It shows that there was no amorphous halo in the FFT, excluding the presence of amorphous phase. Figure 3c shows that more grain boundaries exist in the BFO2 than in the BFO1.

### 3.2. PTCR Effect

**Figure 4:**
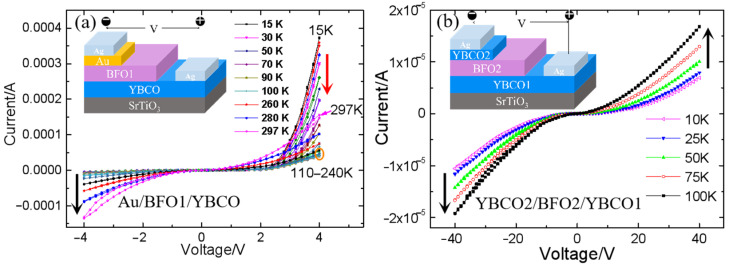
The current versus voltage curves of Au/BFO1/YBCO capacitor (**a**) and YBCO2/BFO2/YBCO1 capacitor (**b**) at different temperatures. The red down arrows illustrate the resistance increases with increasing the temperature, i.e., PTCR. The black vertical arrows indicate the absence of PTCR. The orange ellipse indicates the IV curves between 110–240 K. The inset illustrates the structure of the capacitor and the electrodes.

The PTCR effect was observed in Au/BFO1/YBCO heterostructures, as mentioned in one of our previous reports [24]. Nevertheless, the superconducting transition temperature of YBCO in this study was higher, i.e., 87 K, than that reported previously, i.e., 45 K. We analyzed the source of the PTCR effect and related factors by changing the preparation conditions and the top electrode. Firstly, the PTCR effect appeared in Au/BFO/YBCO; I–V curves of Au/BFO/YBCO in the temperature range of 15 K to 297 K are shown in Figure 4a. Because Au and YBCO have different work functions, there was a rectification effect in Au/BFO/YBCO. When YBCO was a positive voltage, the leakage current was large, and the PTCR effect emerged when the voltage was greater than 2 V. The current at 4 V decreased with a temperature increase from 15 K to 220 K, which meant that the resistance increased with increasing temperature, that is, PTCR. Additionally, PTCR was enhanced with increasing bias voltage, which is consistent with the phenomenon observed in BaTiO_3_ films [17]. Above 220 K, PTCR disappeared. The temperature range of PTCR matched that in Pb(Ti, Zr)O_3_ films [16]. However, the dielectric constant of BFO films did not increase with decreasing temperature in a temperature range of 50 K to 270 K [26], like the BaTiO_3_ films and Pb(Ti, Zr)O_3_ films. Therefore, the PTCR was not caused by the dielectric constant.

The transport current of the Au/BFO1/YBCO capacitor is determined by the characteristics of the two interfaces and the BFO bulk. The PTCR effect was observed in the Au/BFO1/YBCO capacitor at low temperature with a positively biased YBCO bottom electrode. As a comparison, we prepared a YBCO2/BFO2/YBCO1 capacitor and characterized its I–V characteristics at low temperature (as shown in Figure 4b). The top electrode of was changed to YBCO. The two electrodes were symmetrical in the YBCO2/BFO2/YBCO1 capacitor, so the rectification effect was not obvious. PTCR was not found in the YBCO2/BFO/YBCO1 capacitor. It can be seen from the curve that the current increased with an increase of temperature at a specific voltage, be it positive or negative voltage, without PTCR effect. The leakage current in the YBCO2/BFO2/YBCO1 capacitor was significantly smaller. No PTCR was observed even when the voltage increased to 40 V, that is, 10 times that in the Au/BFO1/YBCO capacitor. In most publications about PTCR, the authors suggest that the PTCR results from the grain boundaries of the bulk materials [27,28]. Nevertheless, Figure 3 illustrates that there are more grain boundaries in the BFO2 thin film than in the BFO1 film. Therefore, the grain boundaries are not critical for PTCR in our case. We think that the absence of PTCR in the YBCO2/BFO2/YBCO1 capacitor was caused by the preparation of top YBCO reducing the oxygen vacancy content in BFO, which reduced the leakage current and improved the insulation of BFO. The current in the YBCO2/BFO2/YBCO1 capacitor mainly came from thermal diffusion, so the current passing through the barrier increased with an increase of temperature.

### 3.3. The Oxygen Vacancy Contents

**Figure 5:**
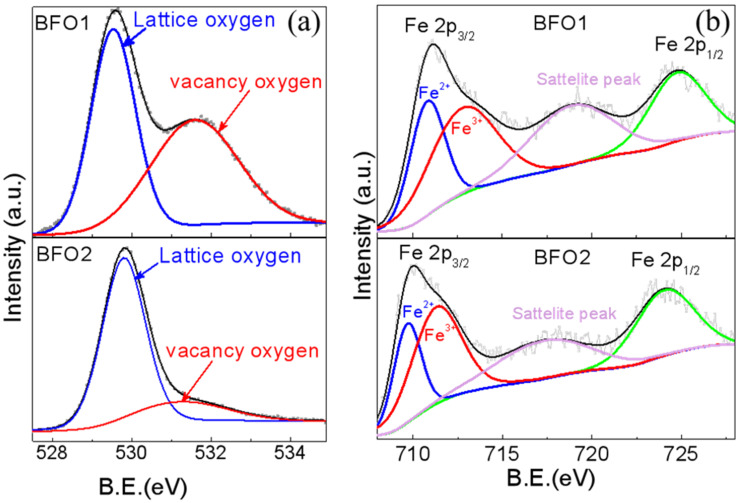
The XPS narrow scan O 1s spectra (**a**) and Fe 2p spectra (**b**) of BFO. The gray lines indicate the experimental curves.

The oxygen vacancy content in the two kinds of BFO capacitors was researched by X-ray photoelectron spectroscopy (XPS). When oxygen vacancies were present in the crystals, the surface chemical states changed, which could be both quantitatively and qualitatively explored by XPS. The XPS spectra for O 1s for BFO1 and BFO2 are presented in Figure 5a. The peak at roughly 529.5 eV (O_I_) in both samples was characteristic of O^2−^; this was associated with the chemical bonding between metal and oxygen [29]. Moreover, another XPS peak was observed at around 531.3 eV (O_II_). This was attributed to the existence of dangling bonds, owing to the formation of surface oxygen vacancies [30]. The relative content of oxygen vacancies could be quantitatively calculated by O_I_/ (O_I_ + O_II_). According to this equation, the calculated oxygen vacancies contents were 49.9% and 20.2% for BFO1 and BFO2, respectively. The longer annealing time of the top electrode YBCO decreased the oxygen vacancy content in BFO. The leakage current also decreased, as shown in Figure 4.

**Figure 6:**
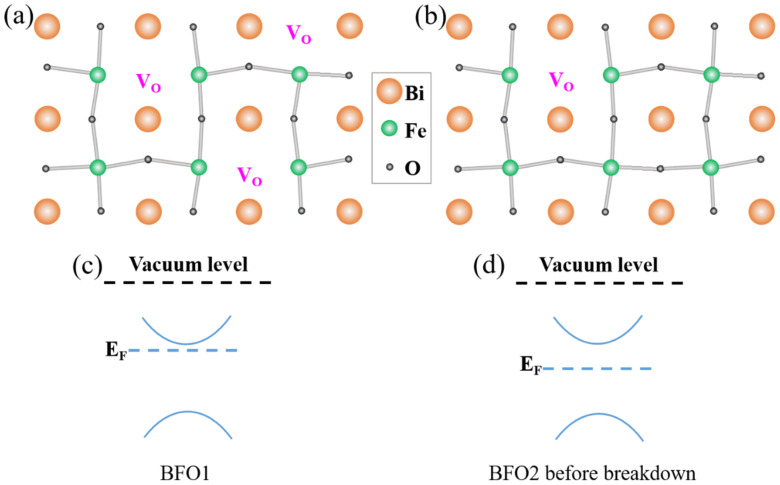
Crystal structure diagrams of BFO1 (**a**) and BFO2 (**b**) with different oxygen contents. The corresponding energy band diagrams of BFO1 (**c**) and BFO2 (**d**).

The same conclusion can also be obtained from the XPS analysis of the Fe 2p peaks. Figure 5b shows that, for BFO, two Fe 2p peaks were observed at 710.2 eV and 724.3 eV, which corresponded to the feature peaks Fe 2p_3/2_ and Fe 2p_1/2_ of Fe^3+^. As there were more oxygen vacancies in BFO1 than in BFO2, the Fe 2p peaks shifted to the higher binding energy, i.e., a Fermi level shift [31,32]. This may be because oxygen vacancies increase the balance between electron density and binding energy [33]. The shift in Fermi level is commonly ascribed to the formation of oxygen vacancies, which would enlarge the equilibrium electron density, and thus, push the Fermi level upward [33]. Fe oxidation could be investigated by XPS. By fitting the peaks for the oxidation state of Fe ions in the XPS spectra, the Fe^3+^ in BFO1 was more than that in BFO2, which revealed the oxidation state in BFO1. The ratios of Fe^2+^ to Fe^3+^ in the BFO1 and BFO2 films were calculated as about 31:69 and 40:60, respectively. This demonstrated more oxygen vacancies in BFO1 compared to BFO2, as shown in Figure 6a,b.

It is well known that oxygen vacancies lead to more electron donors and enhance the donor density of semiconductors, and thus, their conductivity. By increasing the donor density, the transport current in BFO1 could be improved. The Fermi level of BFO1 could also be shifted toward the conduction band (as shown in Figure 6c). The oxygen vacancy content in the BFO2 film of YBCO2/BFO2/YBCO1 capacitor decreased. If the band gap increases, the Fermi level could be shifted toward the middle of the band gap, as shown in Figure 6d.

**Figure 7:**
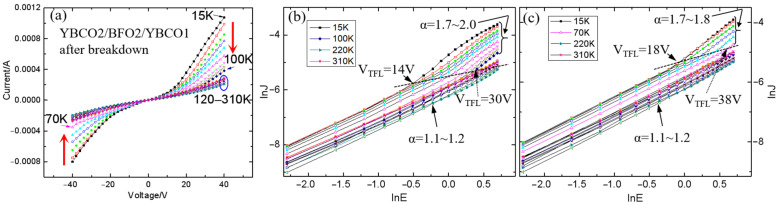
(**a**) Current versus voltage curves of a YBCO2/BFO2/YBCO1 capacitor after breakdown at a temperature ranging from 15 K to 310 K. The corresponding ln*J* versus ln*E* curves of the positive bias voltage (**b**) and the negative bias voltage (**c**).

By comparing the transport properties in Au/BFO1/YBCO and YBCO2/BFO2/YBCO1 capacitors, it was revealed that the PTCR in BFO depends on the oxygen vacancy content. Oxygen vacancies enhanced the charge transport, which meant that PTCR occurred at a lower bias voltage. Therefore, the conductivity of BFO is important for PTCR. To check this result, a 200 V bias voltage was used to breakdown a BFO2 film. As expected, PTCR was observed in the BFO2 film after breakdown, as shown in Figure 7a. After breakdown, the current of BFO at low voltage (<10 V) increased by three orders of magnitude, while the current of BFO at 40 V increased by two orders of magnitude. The Fermi level could be shifted toward the conduction band to improve the conductivity of the BFO film. The PTCR in the BFO2 film after breakdown confirmed that it depended on the Fermi level and bulk conductivity.

### 3.4. PTCR Effect in BFO/YBCO Capacitor after Breakdown

Unlike the Au/BFO1/YBCO capacitor, PTCR was detected in the YBCO2/BFO2/YBCO1 capacitor regardless of whether the YBCO1 bias was positive or negative, as shown in Figure 7a. Moreover, we found that the observed temperature of PTCR depended on T_C_, which was 100 K at positive bias and 70 K at negative bias. It should be noted that under positive bias, the BFO/YBCO1 interface was more critical in the charge transport than the YBCO2/BFO2 interface. The 84 K T_C_ of YBCO1 was higher than that of YBCO2, i.e., 67 K. The higher T_C_ of YBCO1 induced a larger temperature range of PTCR under positive bias. T_C_ dependence of PTCR was also found in Au/BFO/YBCO capacitors. As the YBCO was positively biased, the current of the capacitor was higher than the negative bias, and the current was determined by the bulk BFO and the interface of BFO/YBCO. For the same BFO, a notable PTCR effect was observed below 100 K, as the T_C_ of YBCO was 87 K, as shown in Figure 4a, and below 50 K for 45 K T_C_ YBCO, as reported elsewhere [23]. Therefore, a higher superconducting transition temperature improves the temperature range of PTCR. PTCR depends not only on the bulk conductivity of BFO, but also on the superconducting transition temperature of YBCO. This was illustrated when a cooper-pair was injected into the BFO films. The phenomenon of a superconducting cooper-pair entering a superconductor-semiconductor structure has been reported previously [34,35]. However, more studies are required to understand the relation between the PTCR and the superconductivity of YBCO.

It is essential to understand the leakage current mechanism. The leakage current mechanism of Au/BFO1/YBCO has been discussed in our other articles [36]. Interface-limited Fowler-Nordheim tunneling was dominant in the Au/BFO1/YBCO capacitor at temperatures from 15 K to 80 K. Here, the leakage current mechanism of YBCO2/BFO2/YBCO1 after breakdown was investigated. Figure 7b,c show the plots of ln*J* vs ln*E* for the YBCO2/BFO2/YBCO1 capacitor after breakdown with the YBCO1 electrode, both positively (Figure 7b) and negatively (Figure 7c) biased in a temperature range from 15 K to 310 K. It was observed that at low voltage (<14 V), the curve followed Ohmic behavior with the slope (*α*) value ~ 1. At a higher voltage (>14 V), the curve increased with *α* value ~ 2. The transport behavior of the BFO film resembled the bulk-limited Space-Charge-Limited Currents (SCLC) leakage mechanism. According to the SCLC model, the current density followed Child’s law [37]:

(1) $$J=\frac{9}{8}\varepsilon_{r}\varepsilon_{0}\mu \frac{E^{2}}{L^{3}}$$

where *J* is the leakage current density, *ε_r_* is the relative dielectric constant, *ε*_0_ is the permittivity of free space, *μ* is the carrier mobility, *E* is the applied field and *L* is the film thickness. The key feature of the SCLC mechanism is the square relation between current and voltage.

At a low voltage, electrons from the electrodes or shallow traps near the thin film conduction band were injected into the conduction band to form Ohmic conduction. As the electric field increased, the number of electrons injected into the BFO increased, which exceeded the thermally stimulated density of electrons dominating SCLC conduction. The Child’s law for the SCLC model would be modified for

(2) $$J\propto E^{\alpha },\;\alpha >1.\;$$

where *J* is the leakage current density, *E* is the applied field, *α* is the multiple slopes with exponent.

The transition voltage from Ohmic to modified Child’s law is called the trap limited voltage V_TFL_, which illustrates the voltage required to fill the deep electron traps. We found that V_TFL_ changed from 14 V to 30 V as the temperature increased from 15 K to 100 K for the positively biased YBCO1. As for the negatively biased YBCO1, V_TFL_ changed from 18 V to 38 V with increasing temperature from 15 K to 70 K. This indicates that deeper traps are found with increasing temperature [38,39]. The sample demonstrated PTCR in both Ohmic behavior and SCLC leakage mechanism region Therefore, the bulk nature of BFO thin films played an important role in the PTCR in our case, which was consistent with the previous analysis in this article. It is well known that the more traps, the greater the current. In our case, although the traps in the BFO films were suppressed as the temperature decreased, the current increased at the PTCR temperature range. This shows that bulk conductivity is the basic condition for, but is not the cause of, PTCR. Overall, the source of PTCR was the interface between BFO and YBCO.

## 4. Conclusions

In summary, we found an anomalous electrical transport property in BFO thin films, that is, a positive temperature coefficient effect of resistivity. PTCR was observed in BFO films at low temperature, within which BFO films were in a ferroelectric state. The source of PTCR in the BFO films was analyzed by changing the preparation conditions of the electrode. Firstly, the PTCR in BFO films depends on the bulk conductivity. The YBCO/BFO/YBCO heterostructure did not show any PTCR effect, but a notable PTCR was observed after the YBCO/BFO/YBCO had been broken down. After breakdown, the conductivity of the BFO film at the low voltage (<10 V) increased by three orders of magnitude, making it equivalent to that of BFO in a Au/BFO/YBCO capacitor. Secondly, the temperature range of PTCR in BFO films may be related to the superconducting transition temperature. For the Au/BFO/YBCO heterostructures, a notable PTCR effect was observed below 100 K and 50 K, as the superconducting transition temperature of YBCO is 87 K and 45 K, respectively. For the YBCO2/BFO/YBCO1 heterostructures, a notable PTCR effect was observed below 70 K and 100 K, as the superconducting transition temperature of YBCO2 and YBCO1 is 67 K and 84 K, respectively. Therefore, higher superconducting transition temperature increases the temperature range of PTCR. The conduction mechanism of YBCO/BFO/YBCO capacitors after breakdown was analyzed. It was found that it was more in line with the SCLC conduction mechanism. The sample exhibited PTCR in both low-voltage Ohmic behavior and high-voltage SCLC regions. The importance of superconductivity on PTCR was confirmed. However, more research is required to understand the relationship between PTCR and superconductivity. Our findings provide a new film material and research direction for the application of PTCR.

## Data Availability

Data presented in this study are available on request from the first author.
